# Emerging applications of long-read sequencing in hematological malignancies: highlights from the 2025 ASH annual meeting

**DOI:** 10.1186/s40364-026-00942-y

**Published:** 2026-06-07

**Authors:** Boyan Liu, Yuan Jiang, Bihuan Zhao, Hong-Hu Zhu

**Affiliations:** 1 Department of Hematology, Beijing Chao-Yang Hospital, Capital Medical University, Beijing, 100020 China; 2 Chinese Institute for Medical Research, Capital Medical University, Beijing, 100069 China; 3 Institute for Cancer, Chinese Institutes for Medical Research (CIMR), Beijing, 100069 China

**Keywords:** Long-read sequencing, Hematological malignancies, Rapid diagnostics, Genetic classification

## Abstract

Long-read sequencing (LRS) is rapidly advancing and demonstrates considerable promise in hematological malignancies. At the 2025 ASH meeting, numerous studies highlighted advances in applying LRS across diverse hematological malignancies. Rapid LRS-based whole-genome sequencing and adaptive sampling enable real-time subtype assignment, detection of SNVs, CNVs, and gene fusions, as well as methylation-based classification. By spanning repetitive and structurally complex regions, LRS enhances genetic subtyping and uncovers clinically relevant structural variants often missed by short-read technologies. Full-length and single-cell RNA sequencing further resolve isoforms, splicing programs, and fusion transcripts within their clonal contexts, supporting precision therapy and refined risk stratification. As accuracy improves and costs decline, LRS is poised for broader integration into routine clinical diagnostics.

## To the Editor

**Fig. 1:**
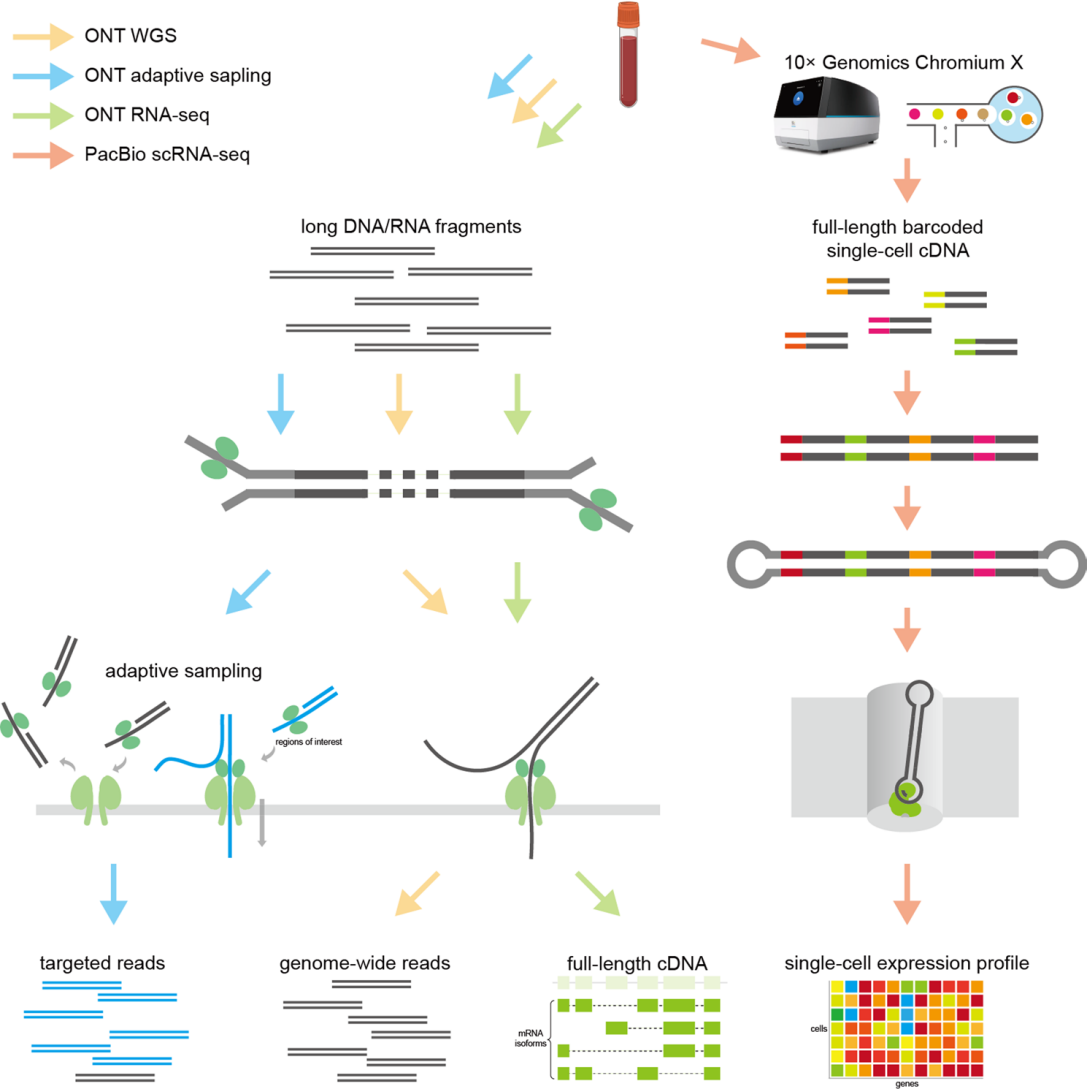
The brief flowchart of the platforms and methods covered in this review. ONT, Oxford Nanopore Technologies; PacBio, Pacific Biosciences; scRNA-seq, single-cell RNA sequencing; WGS, whole-genome sequencing

Precise diagnosis and treatment of hematological malignancies increasingly rely on molecular classification and genetic risk stratification. However, conventional approaches, including next-generation sequencing (NGS), have limited ability to resolve complex structural variants (SVs), repetitive regions, and isoform diversity. Long-read sequencing (LRS), represented by Oxford Nanopore Technologies (ONT) and Pacific Biosciences (PacBio), generates kilobase-scale DNA/RNA reads, enabling comprehensive genomic and transcriptomic profiling (Fig. 1). Here, we summarize LRS advances in hematological malignancies presented at the 2025 ASH Annual Meeting (Table 1).

**Table 1:** Advances of long-read sequencing in hematological malignancies in 2025 ASH Annual Meeting

Authors [reference]	Platforms	Methods	Diseases	Highlights	Data Accession
Shah et al. [1]	ONT PromethION	WGS	AML	Rapid genetic diagnosis of AML within one day	Unavailable
Geoffrion et al. [2]	ONT	Adaptive sampling	Pediatric leukemia	Rapid classification for pediatric leukemia	10.1101/2025.10.02.25336569
Achterberg et al. [4]	ONT	Adaptive sampling	Hematological malignancies	Created a methylation data training neural network tool for rapid classification	Unavailable
Capilla-Guerra et al. [5]	ONT PromethION	Adaptive sampling	Acute leukemia	Rapid classification based on neural network methylation	10.1038/s41588-025-02321-z GSE280090
Salmon et al. [6]	ONT MinION	Adaptive sampling	CML	Analysis of typical and atypical BCR::ABL1 rearrangement	Unavailable
Legendre et al. [7]	PacBio Revio	WGS	MM	Analysis of complex immunoglobulin loci translocations in multiple myeloma	Unavailable
Banco et al. [8]	ONT PromethION	Adaptive sampling	Burkitt lymphoma	Limited methylation sites support the classification of Burkitt lymphoma	10.1158/2643-3230.BCD-24-0240 https://portal.gdc.cancer.gov/projects/CGCI-BLGSP
Ma et al. [9]	ONT PromethION	WGS	ALL	Revealed the hidden SVs missed by NGS in ALL	Unavailable
Sekrecki et al. [10]	ONT	Long-read RNA-seq	SF3B1-mut myeloid malignancies and CLL	Analysis of pathogenic UTR alternative splicing	10.1182/blood.2025029972
Thieme et al. [11]	10x GEM-X PacBio Revio	Long-read scRNA-seq	Healthy donor	Constructing a hematopoietic single-cell transcript isoform map	Unavailable
Walter et al. [12]	10x GEM-X PacBio Revio	Long-read scRNA-seq	B-ALL	Analysis of the cellular origin stage of B-ALL fusion genes	Unavailable

Adaptive sampling is a selective sequencing approach that generates targeted reads while also producing low-coverage whole-genome data. ALL, acute lymphoblastic leukemia; AML, acute myeloid leukemia; CLL, chronic lymphocytic leukemia; CML, chronic myeloid leukemia; MM, multiple myeloma; ONT, Oxford Nanopore Technologies; PacBio, Pacific Biosciences; SV, structural variation; UTR, untranslated region; WGS, whole-genome sequencing

## Rapid molecular diagnostics

Genetic and molecular profiling of acute leukemia conventionally requires multiple assays, including karyotyping, fluorescence in situ hybridization, targeted NGS panels, and RNA sequencing, resulting in prolonged turnaround times. In acute myeloid leukemia (AML), LRS-based rapid whole-genome sequencing (WGS) reduces the sample-to-report interval to approximately one day while detecting clinically relevant fusion genes and rearrangements at 50–60× coverage [1].

ONT-based adaptive sampling enables targeted enrichment while preserving low-depth genome-wide coverage. In pediatric leukemia, approximately one hour of sequencing was sufficient to identify large copy number variations (CNVs), with single-nucleotide variants (SNVs) and fusion genes detected within 4.2 and 7.4 h, respectively [2]. Because LRS preserves epigenetic modifications, deep-learning models trained on reference methylomes support rapid methylation-based classification [3]. For example, Lamprey, a deep-learning classifier, robustly distinguished AML subtypes according to WHO/ICC criteria and concurrently provided copy number profiles [4].

A parallel strategy combining conventional and adaptive pores illustrated a tiered “direction first, details later” paradigm. Early methylation signals enabled leukemia subtype assignment and CNV profiling within 2 h, whereas adaptive pores enriched 274 hematological loci to > 100× coverage for comprehensive genetic profiling within 2**–**3 days [5]. In B-acute lymphoblastic leukemia (B-ALL), high-confidence subtype calls were generated within 10 min of sequencing onset, supporting early treatment guidance [2].

## Precision genetic classification

NGS has limited sensitivity for large insertions/duplications, complex translocations, and poorly mappable regions such as immunoglobulin loci. By spanning repetitive and complex rearranged regions, LRS improves genetic subtyping in hematological malignancies.

Using an adaptive sampling hematology-focused panel, studies in chronic myeloid leukemia precisely localized canonical and atypical *BCR::ABL1* breakpoints and additional cryptic SVs, including inserted fragments in karyotypically normal cases [6]. In multiple myeloma (MM), LRS revealed immunoglobulin translocation junctions containing large insertions or unalignable sequences that were undetectable by NGS [7]. LRS-based benchmarking of SV-calling algorithms may further refine MM risk stratification. Targeted long-read approaches also distinguished Burkitt lymphoma from other B-cell lymphomas and resolved epigenetic subtypes, refining subtype classification and risk assessment [8].

In patient-derived xenograft models of B-cell precursor ALL, LRS-WGS identified functional SVs missed by NGS, including non-coding insertions in *MEF2D* and *PAX5* intragenic amplifications, which were associated with glucocorticoid resistance and prognostic heterogeneity [9]. In *MTAP*-deleted models, synthetic-lethal sensitivity to combined MTA and PRMT5 inhibition further underscored the therapeutic value of LRS-defined structural alterations [9].

## Full-length transcriptomic profiling

RNA-LRS directly characterizes full-length isoforms, splice events, and fusion transcripts. In *SF3B1*-mutant myeloid malignancies and chronic lymphocytic leukemia, ONT full-length RNA sequencing revealed complex *DCAF16* alternative splicing, generating novel long non-coding transcripts and enhancing *DCAF16* expression [10]. *DCAF16*-based protein degraders showed preferential activity in *SF3B1*-mutant cell lines and primary samples, suggesting therapeutic relevance.

PacBio sequencing can be integrated with 10x Genomics single-cell library preparation, enabling long-read single-cell RNA sequencing (scRNA-seq). Thieme et al. generated a single-cell isoform atlas across hematopoiesis and developed dedicated analytical pipelines [11]. They characterized canonical splicing events and revealed a shift in *BCL2L1* isoforms from anti-apoptotic to pro-apoptotic variants, providing a framework for understanding isoform dysregulation in myelodysplastic neoplasms and AML. In B-ALL, long-read scRNA-seq comprehensively detected fusion transcripts arising from chromosomal rearrangements or aberrant splicing and resolved their clonal distribution in pre-B-cell subsets [12].

In summary, LRS is rapidly advancing the molecular diagnosis of hematological malignancies by enabling integrated detection of structural, epigenetic, and transcript isoform abnormalities. However, key limitations persist, including reduced sensitivity for low-VAF somatic variants, requirements for high-quality, high-input DNA and adequate tumor burden, small validation cohorts, and substantial cost and bioinformatic demands [1, 2]. Currently, a hybrid strategy**—**LRS for rapid SV and fusion-gene resolution combined with NGS or orthogonal assays for sensitive small-variant detection**—**may represent the most practical clinical solution.

## Data Availability

No datasets were generated or analysed during the current study.

## Abbreviations

AML: Acute myeloid leukemia
CNV: Copy number variant
LRS: Long-read sequencing
MM: Multiple myeloma
NGS: Next-generation sequencing
ONT: Oxford nanopore technologies
PacBio: Pacific biosciences
scRNA-seq: Single-cell RNA sequencing
SNV: Single-nucleotide variant
WGS: Whole-genome sequencing

## References

[1] Shah P, Deharvengt S, Wainman L, Green D, Spracklin S, Balcome S, et al. Single day long read whole genome sequencing for acute myeloid leukemia. Blood. 2025;146(Supplement 1):5286–5286. 10.1182/blood-2025-5286.

[2] Geoffrion N, Lawruk-Desjardins C, Langlois S, Aleman Alvarado M, Dreyer N, Carrier A, et al. Comprehensive and rapid detection of genomic alterations in pediatric leukemias using whole-genome sequencing with adaptive sampling. Blood. 2025;146(Supplement 1):4337–4337. 10.1182/blood-2025-4337.

[3] Steinicke TL, Benfatto S, Capilla-Guerra MR, Monteleone AB, Young JH, Shankar S, et al. Rapid epigenomic classification of acute leukemia. Nat Genet. 2025 Sep;22. 10.1038/s41588-025-. 02321-z PubMed PMID: 40983754. 10.1038/s41588-025-02321-z PMC12513838 40983754

[4] Achterberg T, De Ruijter E, Van Tuil M, Waanders E, Goemans B, Calkoen F, et al. Rapid DNA-methylation based classification of hematological malignancies. Blood. 2025;146(Supplement 1):119–119. 10.1182/blood-2025-119.

[5] Capilla-Guerra M, Young J, Steinicke T, Benfatto S, Hovestadt V, Chen E, et al. Rapid diagnosis of acute leukemia with integrated epigenetic and genetic profiling. Blood. 2025;146(Supplement 1):937–937. 10.1182/blood-2025-937.

[6] Salmon M, Rinke J, Ernst T, Tapper W, Hochhaus A, White H, et al. Nanopore sequencing to detect BCR::ABL1 and associated genomic rearrangements in CML. Blood. 2025;146(Supplement 1):1995–1995. 10.1182/blood-2025-1995.

[7] Legendre C, Turner B, Kyman S, Enriquez D, Jepsen W, Truong S, et al. Transitioning from FISH to NGS for immunoglobulin translocation detection in multiple myeloma. Blood. 2025;146(Supplement 1):5724–5724. 10.1182/blood-2025-5724.

[8] Banco G, Dreval K, Hilton L, Scott D, Trimble MJ, Lee AHY, et al. Evaluating targeted long-read sequencing as a diagnostic tool for burkitt lymphoma. Blood. 2025;146(Supplement 1):3547–3547. 10.1182/blood-2025-3547.

[9] Ma X, Jiang M, Wang J, Mi JQ, Wong JWH, Jing D. Long-read profiling of structural variants reveals mechanisms of chemo-resistance and prognostic heterogeneity in acute lymphoblastic leukemia. Blood. 2025;146(Supplement 1):1585–1585. 10.1182/blood-2025-1585.

[10] Sekrecki M, Sekrecka A, Lattupally R, Le K, Cao X, Chen V, et al. Novel therapeutics for SF3B1 mutant cancers which exploit the missplicing of DCAF16. Blood. 2025;146(Supplement 1):1474–1474. 10.1182/blood-2025-1474.

[11] Thieme E, Arriaga-Gomez E, Lee S, Setty M. From co-occurrence to mutual exclusivity: long read single-cell rnaseq atlas of healthy human bone marrow unveils RNA isoform landscape. Blood. 2025;146(Supplement 1):6133–6133. 10.1182/blood-2025-6133.

[12] Walter W, Kern W, Stengel A. Long-read single-cell isoform sequencing for cell type-specific detection of genomic rearrangement-dependent and -independent fusion transcripts. Blood. 2025;146(Supplement 1):6117–6117. 10.1182/blood-2025-6117.

